# Long-Term Effect of Antibodies against Infused Alpha-Galactosidase A in Fabry Disease on Plasma and Urinary (lyso)Gb3 Reduction and Treatment Outcome

**DOI:** 10.1371/journal.pone.0047805

**Published:** 2012-10-19

**Authors:** Saskia M. Rombach, Johannes M. F. G. Aerts, Ben J. H. M. Poorthuis, Johanna E. M. Groener, Wilma Donker-Koopman, Erik Hendriks, Mina Mirzaian, Sijmen Kuiper, Frits A. Wijburg, Carla E. M. Hollak, Gabor E. Linthorst

**Affiliations:** 1 Department of Endocrinology and Metabolism, Division of Internal Medicine, Academic Medical Center, Amsterdam, The Netherlands; 2 Department of Medical Biochemistry, Academic Medical Center, Amsterdam, The Netherlands; 3 Department of Pediatrics, Academic Medical Center, Amsterdam, The Netherlands; UAE University, Faculty of Medicine & Health Sciences, United Arab Emirates

## Abstract

**Introduction:**

Enzyme replacement therapy (ERT) with alpha-Galactosidase A (aGal A) may cause antibody (AB) formation against aGal A in males with Fabry disease (FD). Anti agalsidase ABs negatively influence globotriaosylceramide (Gb3) reduction. We investigated the impact of agalsidase AB on Gb3 and lysoGb3 and clinical outcome in Fabry patients on ERT.

**Methods:**

Adult male and female patients on ERT for at least one year were included. Urinary Gb3 was measured by HPLC, plasma lysoGb3 by LC-ESI-MS/MS and AB with a neutralization assay.

**Results:**

Of the 59 patients evaluable patients, 0/30 females and 17/29 males developed anti-agalsidase antibodies (AB+). Only 3/17 males had transient (low) titers (tolerized). All AB+ patients developed antibodies during the first year of treatment. Change of agalsidase preparation (or dose) did not induce antibody formation. AB+ males had significant less decline in plasma lysoGb3 compared to AB− males (p = 0.04). Urinary Gb3 levels decreased markedly in AB− but remained comparable to baseline in AB+ males (p<0.01). (Lyso)Gb3 reduction in plasma and urine on ERT was correlated with LVmass reduction in females and development white matter lesions and stroke.

**Conclusion:**

In male patients antibodies against aGal A remained present up to 10 years of ERT. The presence of these antibodies is associated with a less robust decrease in plasma lysoGb3 and a profound negative effect on urinary Gb3 reduction, which may reflect worse treatment outcome.

## Introduction

The X-linked lysosomal storage disorder Fabry disease is caused by deficiency of the hydrolase alfa-galactosidase A. Due to this defect glycosphingolipids, primarily globotriaosylceramide (Gb3, also known as ceramidetrihexoside) accumulate in various cells of the body. Gb3 storage is the primary event that ultimately results in clinical symptoms that start at childhood and comprise acroparesthesia, anhidrosis, and angiokeratoma. At adult age, renal, cardiac, and cerebrovascular involvement determine the reduced life expectancy seen in this disease [1]. Both hemizygous males and heterozygous females can be affected by Fabry disease, though in females the disease course is in general milder and more protracted.

Increased levels of Gb3 can be demonstrated in organs, plasma, and urine , especially in affected Fabry males. In contrast, the majority of female Fabry patients have normal Gb3 levels in blood although most have increased levels of Gb3 in urine [2].

Clinical trials have demonstrated that biweekly infusions (enzyme replacement therapy or ERT) with two distinct aGal A preparations reduce the Gb3 content in kidney, heart and skin [3], [4]. Plasma Gb3 levels decline to normal values within 3 months, while urinary Gb3 clearance is less prominent [3]. Since repeated organ biopsies are not feasible to evaluate treatment efficacy, serial measurement of plasma and urine Gb3 as a marker for treatment efficacy is advised, though the exact relevance for monitoring therapeutic efficacy of ERT has not been elucidated yet.

Emergence of antibodies towards the infused enzyme is commonly observed in Fabry males and has a negative impact on urinary Gb3 clearance [5]. A similar observation was made in skin, with recurrence of Gb3 accumulation in patients with high antibody titers [6], [7]. To study the clinical impact of these antibodies on a biochemical level, renal function was used as outcome measurement in one study, in combination with clinical events (e.g. progression of disease) [6]. Analysis of patients who participated in two clinical trials and for whom long term outcome of 5 years of ERT was available did not demonstrate a difference in renal function or clinical events, but this was compared only for different titer subgroups (high, intermediate, low and no antibodies) and no direct comparison between patients with and without antibodies was made [6].

The negative effect of antibodies on Gb3 clearance is influenced by agalsidase dose. Patients who switched from agalsidase alfa or beta 0,2 mg/kg to agalsidase beta 1,0 mg/kg demonstrated an additional decrease in plasma Gb3 in AB+ patients 12 months after switch[8].

Detailed analysis of the effect of long term ERT on plasma Gb3 or urinary Gb3 in relation to the presence agalsidase antibodies is lacking. Recently de-acylated Gb3 (globotriaosylsphingosine, or lysoGb3), was shown to be highly increased in plasma of patients with Fabry disease, its relative elevation exceeding markedly that of Gb3 [9]. Plasma lysoGb3 proved to be an independent risk factor for white matter lesions in males and left ventricular hypertrophy in females [10] and was correlated with other markers of renal injury [11]. Lifetime exposure to plasma lysoGb3 tended to correlate with disease severity [10]. Enzyme replacement therapy reduced lysoGb3 levels during the first 12 months and these reductions were influenced by dose and the presence of antibodies, with higher doses resulting in a more robust lysoGb3 reduction [12] similar to the effects seen for Gb3 [8]. As lysoGb3 levels in females are more often increased than plasma Gb3 values, lysoGb3 may be a more attractive biochemical marker than Gb3 for monitoring the effect of ERT.

The aim of the current study was to analyse the impact of antibodies on long-term biochemical, and clinical outcome of ERT in Dutch Fabry patients.

## Methods

### Patients

Of the entire cohort of 70 adult Dutch Fabry patients only those presenting with classical manifestations of Fabry disease and treated with either agalsidase alfa or beta (ERT) for at least one year were included (n = 59). Classical Fabry disease is defined by the presence of characteristic symptoms and elevated plasma lysoGb3 and/or Gb3 in hemizygotes, and in case of heterozygotes, having a mutation known to be associated with a classical phenotype through literature or a male family member with classical symptoms [12]. Analyses were conducted until December 2010 or August 2010 in patients with dose-reductions because of a shortage of agalsidase beta [13]. In all patients plasma lysoGb3, Gb3 and urinary Gb3 were measured at baseline and for most patients also in subsequent years. In addition, antibody status was determined 6 months after start of ERT and yearly after that. Patients were treated with agalsidase alfa 0.2 mg/kg/2 weeks, with agalsidase beta 0.2 mg/kg/2 weeks as part of a previous clinical trial [14] or with agalsidase beta 1.0 mg/kg/2 weeks. Two groups were analysed: all patients and a subgroup consisting of males treated for more than five years. The latter group consisted of 8 AB+ and 9 AB− males.

Clinical evaluations were performed yearly and consisted of neurological, cardiac, and renal evaluations. The estimated glomerular filtration rate (eGFR) was calculated according to the abbreviated MDRD formula [15]. Left Ventricular Mass (LVM) was assessed by echocardiography and calculated according to Devereux [16]. Presence of (new) cerebral White Matter Lesions (WML) was determined with MRI by experienced neuro-radiologists. A complication was defined as any cardiac complication, stroke, or end stage renal disease. Cardiac complications were defined as: onset of atrial fibrillation, onset of any other rhythm disturbance needing hospitalization, pacemaker, or implantable cardiac defibrillator (ICD) implantation, cardiac congestion for which hospital admittance was needed, myocardial infarction, percutaneous coronary intervention, or coronary artery bypass graft. ESRD was defined as CKD stage 5 (a GFR<15 ml/min/1.73 m^2^), starting dialysis or renal transplantation. The occurrence of a clinical event was recorded and correlated with antibody status in patients treated for five years or more. A summary of the baseline characteristics of the patients included in the study is given in Table 1.

**Table 1:** Baseline characteristics of all adult classic patients treated >1 year.

	AB+males	AB−males	AB males who tolerized	Females
Number of patients	14	12	3	30
Age at start of ERT (median, range)*	41.0 (18.1–58.5)	22.8 (17.0–65.3)	41.8 (27.2–42.7)	45.6 (15.9–71.5)
LysoGb3 nmol/L (healthy controls 0.3–0.5)	85 (31–124)	74 (20–160)	102 (100–112)	8 (2.7–24)
Plasma Gb3 umol/L (healthy controls 0.45–2.46)	4.2 (1.8–7.1)	3.8 (1.5–5.7)	4.5 (3.1–5.9)	1.5 (0.5–2.8)
Urinary Gb3 nmol/24 h (healthy controls 18–90)	1885 (608–4691)	1928 (250–4494)	1689 (911–2905)	343 (20–2009)
eGFR	84.1 (24.6–130.1)	128.1 (35.5–208.3)	100.8 (38.0–151.1)	98.7 (44.7–180.5)
LVmass (g/m^2.7^ per year)	53.3 (31.1–77.3)	44.5 (25.4–67.7)	53.6 (29.2–66.4)	44.1 (25.5–120.0)
Presence of white matter lesions	9/12	4/10	2/3	20/28
History of a complication	4/14	1/12	0/3	3/30

* 2 males and 4 females started ERT during childhood.

### Consent and IRB approval

Analysis of glycolipids and antibodies is part of standard of care in the treatment of Fabry disease in our expert center. A waiver from the hospital's institutional review board stating was obtained, stating that this analysis does not apply to the Medical Research Involving Human Subjects ACT (WMO) and that an official IRB approval by the institution is not required. All patients gave written consent for treatment, storage and analysis of blood samples.

### Biochemical analyses

Yearly Gb3, lysoGb3 and antibody assessments were performed. Antibody (AB) analysis was performed by incubating plasma of patients with 1 ng agalsidase alfa or beta (depending on the enzyme preparation the patient was treated with). The amount of plasma needed to neutralize 50% of the enzymatic activity of 1 ng agalsidase alfa or beta was determined by titration. A sample was considered anti-agalsidase IgG negative if a sample demonstrated less than 50% neutralization at 5 µl plasma input. Previously, it was already demonstrated that the amount of neutralizing activity directly correlates with antibody titer [5]. In addition, that study also demonstrated that removal of IgG abolished the inhibitory effect of anti-agalsidase positive plasma.

### Globotriaosylceramide (Gb3) and globotriaosylsphingosine (lyso-Gb3) analysis in plasma

Blood sampling in EDTA was performed for measurement of Gb3 and lyso-Gb3. Blood samples were centrifuged for 10 minutes at 3000 rpm to obtain plasma. Plasma was stored at −20°C until use. Gb3 analysis was performed by using high-performance liquid chromatography essentially as described by Groener et al [17]. Fifty µl of plasma was used and lipids were extracted according to Bligh and Dyer [18]. As internal standard C17 sphinganine was used. The intra-and interassay coefficient of variation is 4% and 14% respectively. Lyso-Gb3 concentrations in plasma samples were measured using 5,6,7,8,9 ^13^C_5_-lysoGb3 as internal standard, extraction by a modification of the method of Bligh and Dyer, and LC-ESI-MS/MS (Gold et al, submitted). The intra- and interassay coefficient of variation is 3.5% and 6.3%, respectively.

### Gb3 analysis in urine

A 50 mL urine sample was obtained from a 24 hour urine collection and stored at −20°C until Gb3 analysis was performed essentially as described for plasma with a slight modification. In short, 400 µl of urine was used and lipids were extracted according to Bligh and Dyer [19]. Instead of water, 20 mM phosphate buffer (pH 8.0) was used in the phase separation.

### Statistical analysis

Results are presented as median (range) or mean±standard deviation (SD). Chi-square was used for comparison of proportions and Mann-Whitney U to compare data between two independent groups and the Kruskal-Wallis test for more than 2 groups. Spearman's rho was used for correlation of antibody concentration (titer) and changes in (lyso)Gb3. For comparison of years of follow-up to baseline, the Wilcoxon-sign rank test was applied. Repeated measurements were used for analysis of LVMass and renal function and Cox regression to analyze time to first white matter lesions/stroke during ERT and the association with (lyso) Gb3 level.

Results were considered to be statistically significant when p-values were <0.05.

## Results

### Antibody status

Of the 59 adult patients on ERT with one year of follow-up, 29 males and 30 females received at least one year of ERT (table 1). None of the 30 female patients developed antibodies during treatment. Of the males, 17 developed antibodies, whereas 12 did not (table 2). Treatment with agalsidase alfa was associated with a lower incidence of antibody formation as compared to agalsidase beta (5/14 on agalsidase alfa vs 12/15 on agalsidase beta, p = 0.025) but in the agalsidase beta group two different dosages were used (0.2 and 1,0 mg/kg). Prevalence of antibody formation between patients treated with 0.2 mg/kg agalsidase alfa or beta did not differ significantly (5/9 versus 2/5 respectively, p = 0.18). All patients who developed antibodies did so within the first year after start of ERT. Treatment alterations after one year of treatment, i.e. an increase in dose (agalsidase beta 0.2 to 1.0 mg/kg) or a switch from agalsidase alfa to agalsidase beta, did not induce antibody formation in those who were negative after one year of treatment (data not shown).

**Table 2:** Antibody formation in all adult male patients and the treatment received in the first year *).

		Treatment		
		Agalsidase alfa	Agalsidase beta**)	Total
Antibodies	AB neg	9	3	12
	AB pos	5	12	17
Total		14	15	29

* including those who tolerized (n = 3)

** A significant proportion (n = 5) of patients on agalsidase beta received 0,2 mg/kg as part of a clinical trial during the first years of treatment.

In three patients AB's disappeared (i.e. these patients tolerized). These three patients had their highest titer already within the first year of treatment, and two of these three had the lowest titer of the 17 AB positive patients. In the other 14 patients, a maximum titer was achieved at later time points and no tolerization was observed (non-tolerized AB+ patients).

### Plasma lysoGb3 and Gb3

**Figure 1:**
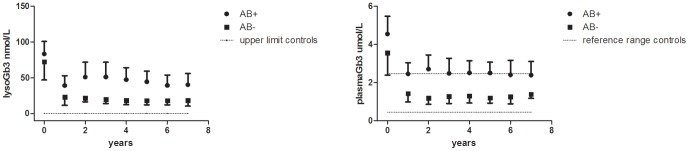
Plasma lysoGb3 and Gb3 in Fabry males. LysoGb3 (A) and plasma Gb3 (B) in 23 classic Fabry males, treated for at least one year (AB+ group n = 12, AB− group n = 11), depicting mean and 95% confidence intervals.

**Figure 2:**
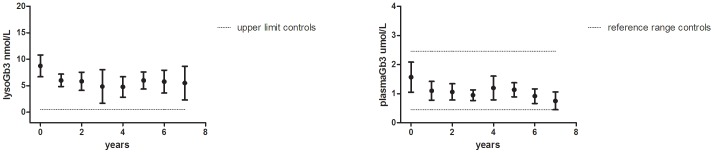
Plasma lysoGb3 and Gb3 in Fabry females. Plasma lysoGb3 in 27 (A) and Gb3 (B) in 28 Fabry females, treated for at least one year, depicting mean and 95% CI.

Of the 29 males, 26 males had follow-up plasma Gb3 and lysoGb3 measurements after one year. In all classic adult male patients levels of plasma lysoGb3 decreased, but this decrease was more robust in male patients without antibodies. Baseline lysoGb3 in both groups was not different (p = 0.33). The 3 patients who tolerized were not included in the subanalysis by AB status; data of the 12 AB+ male patients were compared to 11 AB− male patients. The males in the AB+ group were treated for a median time of 7.6 (1.3–10.2) years, the AB- group for 6.1 (1.2–8.1) years, p = 0.24). At baseline, lysoGb3 in both groups was not different (p = 0.41). In the first year, lysoGb3 decreased in both the AB+ group (from 81 (31–124) to 36 (15–80) nmol/L, n = 12) as well as in the AB− group (from 73 (20–103) to 24 (6–47) nmol/L, n = 11). However during follow-up, lysoGb3 remained elevated (reference range in healthy controls: 0.3–0.5 nM) and were higher in the AB+ group compared to the AB− group (P<0.043, year 1 through 6 compared to baseline, fig 1a). Analysis of plasma Gb3 in the same patients demonstrated a similar pattern (fig 1b), but changes were less pronounced as Gb3 levels are less increased at baseline as compared to lysoGb3. Gb3 levels remained higher in het AB− group compared to the AB+ group (p<0.02). Also in females with follow-up data, both lysoGb3 (from 8 (2.7–24) to 5 (2–13), n = 27) and Gb3 (from 1.6 (0.5–2.8) to 1.1 (0.5–1.8) n = 28) declined after the first year and remained decreased after 6 years (p<0.03) (figure 2a and b) (range 2.3–11.4 nM), even though lysoGb3 did not normalize and remained elevated as compared to normal controls (0.3–0.5 nM).

### Urinary Gb3

**Figure 3:**
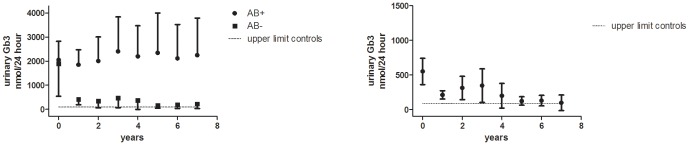
Urinary Gb3 in males and females. **A.** Urinary Gb3 in classic Fabry males, treated for at least one year (AB+ group n = 11, AB− group n = 11). **B.** Urinary Gb3 in classic Fabry females treated for at least one year (n = 28), depicting mean and 95% confidence interval. The dotted line denotes upper limit of normal in healthy controls: 18–90 nmol/24 uur.

Male patients with antibodies and those without showed a clear difference in urinary Gb3 reductions. Mean urinary Gb3 decreased from 1818 (250–4494) to 390 (68–735) nmol/24 h (p = 0.003) in all antibody negative males (n = 11) with follow-up after the first year and remained decreased after 6 years of ERT compared to baseline (range 19–387 nmol/24 hours, p = 0.028), to normal or near normal values, (healthy controls: 18–90 nmol/24 hours, fig.3a). Mean urinary Gb3 levels in patients with antibodies in patients treated for 1 year or more with follow-up (N = 11) did not decrease (from 1812 (608–4691) to 1728 (212–3619) after 1 year, p = 0.79), and remained elevated for up to 8 years of treatment (216–4547 nmol/24 hours, fig 3a). In the 25 females with urinary Gb3 data, urinary Gb3 decreased from 379 (20–2009) to 173 (17–620) nmol/24 h after the first year and remained decreased after 6 years of follow-up (28–339 nmol/24 hours, p = 0.005, fig 3b).

### Effect of dose and change of preparation

In the 23 males with follow-up data (excluding the patients who tolerized), lysoGb3, plasma Gb3 and urinary Gb3 were comparable at baseline, irrespective of preparation or dose received (see table 3). After the first year of treatment, in males treated with agalsidase alfa or beta 0.2 mg/kg/2 weeks, the decrease of lysoGb3 was less pronounced compared to the AB+ group treated with 1.0 mg/kg/2 weeks (p<0.03) and the AB− group (p<0.02). Also the decline of plasma and urinary Gb3 in the AB+ group treated with agalsidase alfa 0.2 mg/kg/2 weeks was less pronounced compared to the AB+ group treated with 1.0 mg/kg (p<0.05) and AB− group (p<0.01).

**Table 3:** Plasma and urinary lipids in male Fabry patients *.

Total	AB+R0.2	AB+F0.2	AB+F1.0	AB− total	*p-value (comparing all groups)*
Number	4	4	4	11	
LysoGb3 baseline (nmol/L)	91 (70–119)	79 (31–114)	76 (53–124)	73	0.68
LysoGb3 1 year ERT	65 (38–80)	36 (30–48)	17.5 (15–23)	24 (6–47)	0.003
Plasma Gb3 baseline (umol/L)	4.7 (4.0–7.1)	3.6 (1.8–5.3)	5.3(3.1–6.1)	3.7 (1.5–5.7)	0.14
Plasma Gb3 1 year ERT	3.6 (3.5–4.1)	2.1 (1.5–3.4)	2.0 (1.9–2.1)	1.2 (0.6–1.5)	0.009
Urinary Gb3 baseline (nmol/24 h)	1786 (1362–2778)	2130 (608–2979)	1790 (669–1812)	1818 (250-4494)	0.81
Urinary Gb3 1 year ERT	2442 (1714–2720)	1748 (1376–3619)	824 (212–1656)	390 (68–735)	0.003

* ) including males with follow-up measurements, without the 3 males who tolerized.

In males with antibodies and treated with agalsidase beta 1.0 mg/kg/2 weeks, the decrease in lysoGb3 within the initial first year was comparable to the AB− group (p = 0.43) as well as the decrease in plasma Gb3 (p = 0.17) and urinary Gb3 (p = 0.24).

After at least one year of treatment, six AB+ males switched to a dose of 1.0 mg/kg/2 weeks. In these males, lysoGb3 declined additionally (median −13 nmol/L, range −33, −9, p = 0.028) one year after switch. Similarly, following switch plasma Gb3 declined additionally (median −0.86 (range −1.1, 0 µmol/L, p = 0.028), while urinary Gb3 declined only in a subset of males (median −216, range −1072, 576 nmol/24 hours, p = 0.75). Despite this additional decrease in lysoGb3, plasma and urinary Gb3 levels in AB+ patients who switched to agalsidase beta 1.0 mg/kg/2 weeks, these levels remained higher as compared to AB− patients (p<0.02).

### Correlation with clinical events

To study the relation of antibodies with clinical outcome, males with antibodies were compared to males without antibodies, the 3 tolerized patients were excluded. AB+ patients were older and had more advanced disease at start of treatment, as compared to antibody negative males (see table 1). This hampers the comparison, as older patients with more advanced disease are more likely to demonstrate disease progression. Selecting only those patients treated with at least five years of ERT (8 AB+ and 9 AB− patients), again males with antibodies were older than the patients without antibodies. In this subgroup, a tendency towards a higher incidence of new complications was seen (4/9 in AB+ vs 1/8 in AB− patients), but this was not statistically significant (p = 0.18). No difference was seen in long term outcome of ERT between these small groups of male patients with regard to renal function, LVmass and the occurrence of new white matter lesions on cerebral MRI.

### Clinical relevance of changes in lysoGb3 and Gb3 in plasma and urine

**Figure 4:**
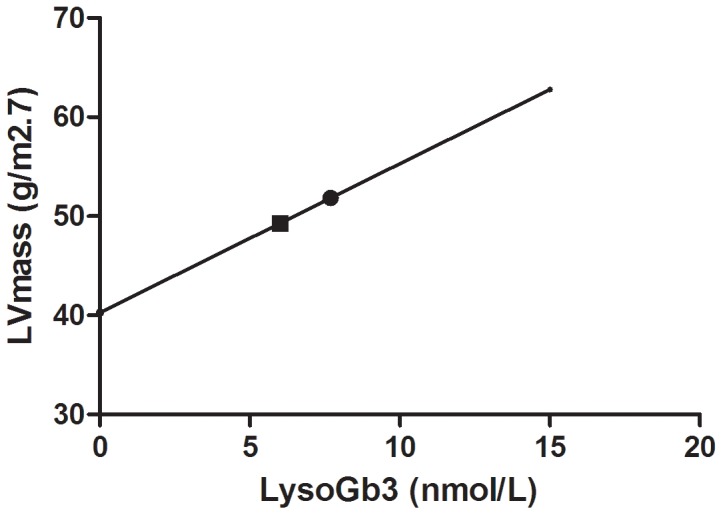
LVMass reduction and LysoGb3. LVMass decreases as lysoGb3 decreases. The dot represents the mean lysoGb3 and mean LVmass in females at start of ERT treatment, the square depicts mean lysoGb3 one year after start of ERT.

In females (n = 29), 1 nM lysoGb3 decrease was correlated with 1.5 g/m^2.7^ decrease in LVmass (p<0.001) independent of age or years of treatment (see figure 4). For plasma Gb3, 1 µM decrease of plasma Gb3 was correlated to 6.1 g/m^2.7^ decrease in LVmass (p = 0.005), for urinary Gb3 100 nmol/24 h decrease was associated with 1.3 g/m^2.7^ decrease in LVmass (p<0.001) independent of age.

**Figure 5:**
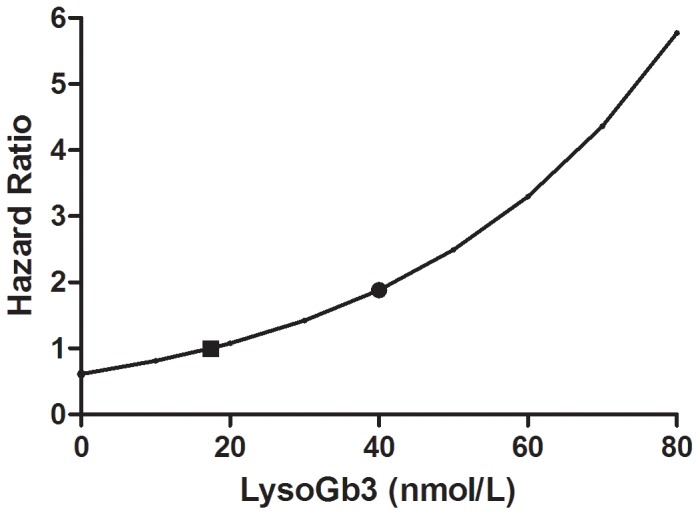
Risk to develop white matter lesions and LysoGb3. The hazard ratio to develop a (additional) white matter lesion or stroke decreases as the absolute value of lysoGb3 decreases. The dot represents mean lysoGb3 for males and females at start of ERT. The square depicts mean lysoGb3 level one year after start of ERT.

For both males and females, reductions in plasma lysoGb3, plasma Gb3 and urinary Gb3 within the first year after start of ERT predicted the hazard of developing white matter lesions and stroke during ERT, while age and gender did not. The hazard ratio for developing white matter lesions and stroke was 0.74 (95%: 0.60–0.90) per 10 nM lysoGb3 decrease) during treatment (p = 0.009, see figure 5)). A reduction of 1 µmol/L plasma Gb3 decreased the hazard ratio to develop white matter lesions or stroke during follow-up (HR 0.54, 95% CI: 0.34–0.87) (p = 0.012). Similarly, a decrease of 100 nmol/24 h urinary Gb3 reduced this hazard ratio to 0.95 (95% CI: 0.92–0.99), p = 0.018. No correlation between the change of renal function and change of (lyso) Gb3 could be determined.

## Discussion

Here we demonstrate the long term impact of antibody formation in male Fabry patients treated with ERT on clearance of urinary Gb3 and to a lesser extent on plasma Gb3 and lysoGb3 levels. In addition, it is also demonstrated that in both AB+ as AB− males plasma lysoGb3 levels seem to plateau after an initial decrease in the first year of treatment and do not reach normal values. While in those AB+ patients treated with 0.2 mg/kg/2 weeks a switch to a higher dose of 1.0 mg/kg/2 weeks resulted in a further reduction of lyso(Gb3), these levels still remained higher compared to AB− patients. Finally, change of lysoGb3 and plasma and urinary Gb3 levels correlate with LVMass change in females, in males with decrease of LVmass and development of strokes or white matter lesions in both males and females. While it is difficult to predict the consequences of the presence of these antibodies, a potential negative effect on therapeutic effectiveness is likely [2], [19], [20].

Few patients in this study developed complete immune tolerance towards aGal A during prolonged treatment. This finding is consistent with a recent report of the postmarketing surveillance database on agalsidase beta. Of the 571 males treated with agalsidase beta 73% developed antibodies towards the infused enzyme, and only 11% of these tolerized, which is similar to our cohort (3/17, 18%) [21]. The continuous presence of (high) levels antibodies may also have consequences for future alternative therapies. It is conceivable that circulating anti-agalsidase antibodies may exert an effect on future second generation aGal A enzyme preparations, on treatment with chaperones that aim to promote the stability of the patient's endogenous mutated aGal A or even in gene therapeutic approaches. In other lysosomal storage disorders, most notably Pompe disease, several strategies have been proposed to reduce the levels of anti-enzyme antibodies, such as methotrexate or anti-CD20 [22]. In Fabry disease similar studies have been done in the mouse-model, but currently no studies have been performed in man [23]. The findings reported here strengthen the need to study in more detail the clinical consequences of antibody formation and approaches on treatment of patients with antibodies. One such approach may be the use of higher dosages of agalsidase as a previous study and this report demonstrate additional reduction of plasma Gb3 and urinary Gb3 following higher dosages[8]. Another possibility may be to develop immune tolerization regimes.

Previously no correlation between symptoms of Fabry disease and plasma or urinary Gb3 levels was found [2], [19]. which led some to question its use as a biomarker [19], [20]. However, Gb3 is believed to be pivotal in Fabry pathogenesis and clearance of accumulated Gb3 in renal capillary endothelium has been accepted as (surrogate) primary endpoint. In clinical trials reduction of plasma, urinary or organ Gb3 was considered to reflect a positive effect of ERT [3], [4]. In addition, recent studies demonstrate that pre-treatment lysoGb3 levels correlated with some of the clinical features of the disease, such as white matter lesions in males and overall clinical severity and left ventricular mass in females [10]. This observation is extended as lysoGb3 response during ERT reflects change in LVmass and development of white matter lesions. In addition, there is an effect of dose: the higher dose of agalsidase beta led to more robust biochemical responses. This is in line with a study in which a lower dose of agalsidase beta (0.3 mg/kg) led to increases in Gb3 in a subset of patients [24]. A recent observation of switch to a lower dose as a result of a worldwide shortage of agalsidase beta again confirms this finding [13]. These findings once more emphasize that the debate on dose and treatment outcomes needs to be elucidated and deserves additional (clinical) studies.

However, even in those patients who do not develop antibodies, ERT resulted in a far from complete reduction to normal levels of plasma lysoGb3. After 6 years of treatment, plasma lysoGb3 in AB− Fabry patients was on average still 40-fold increased compared to normal values. The fact that decrease in lysoGb3, and in plasma and urinary Gb3 correlates with LVmass reduction upon ERT and influences the hazard of developing white matter lesions, suggests that a more robust decline of these biomarkers is probably associated with a more favourable outcome. Both the phase 4 study with agalsidase beta as well as detailed analyses of the Dutch cohort demonstrate disease progression in many patients, suggestive of a very modest effect of ERT in Fabry disease [14], [25], [26]. This modest treatment effect as well as the influence of end organ damage to the responsiveness of the disease [27], [28] may also explain why this study and that of others [6], [28] fail to demonstrate a clear effect of antibodies on treatment effectiveness. Comparing antibody positive and negative patients with regard to clinical outcome may also be hampered by the fact that patients with residual aGal A activity may not only have a lower risk for developing anti-agalsidase antibodies, but may also have a less severe course of the disease as compared to those without residual enzyme activity. The development of anti-agalsidase antibodies may thus reflect a more severe phenotype of the disease with faster disease progression.

In this study we made some additional observations that may be of practical use in the daily care of patients on ERT. None of the females developed antibodies, which is not surprising as females have substantial residual enzyme activity (given the X-linked nature fo the disease) and the infused recombinant human enzyme is unlikely to be regarded as immunogenic. However, recently another study reported 12% of treated females developed antibodies [21]. This observed difference in antibody formation may be related to the method of antibody detection (neutralization assay vs ELISA/RIP was used in [21]). Our data suggest, that the need to monitor antibodies may be of lesser importance in females, and may be performed less frequently. In addition, in this study all patients who were antibody negative after one year of treatment remained so, irrespective of an increase in dose, or change in enzyme preparation.

Again these observations should be confirmed in larger cohorts, but may be of importance for physicians considering switching preparation or increase the dose.

Our study has some limitations. Despite covering the entire Fabry population of the Netherlands, the total number of analysed individuals remains limited, especially with regard to those on longer duration of treatment (>5 years). Moreover, during the study observation period several patients had their agalsidase dose and/or preparation changed. Our study demonstrated differences in antibody formation between the two treatment preparations, at least when given at their licensed dose. Additional analyses in larger cohorts are necessary to confirm differences between the two agalsidase preparations with regard to antibody formation or effectiveness in reducing Gb3 and lysoGb3. In addition, in this study we used a neutralizing assay to detect antibodies. While a previous study demonstrated that all patients with antibodies had an aGal A neutralizing effect in vitro[5] it is possible that a patient may develop antibodies that do not exhibit neutralizing capacity.

The lack of a uniform assay to detect and quantify agalsidase antibodies hampers direct comparison with other studies. In most studies on antibody status, antibody analyses was performed by the manufacturer of the enzyme preparation used. Subsequently, claims with respect to antibody formation cannot be extrapolated from one preparation to the other. This emphasizes the need for standardization of anti agalsidase assays and the results of a standardization initiative is eagerly awaited [29].

In conclusion, we demonstrated a negative effect of anti-agalsidase antibodies in male Fabry patients on Gb3 and lysoGb3 levels in plasma and particularly profoundly that of Gb3 in urine. Change of (lyso)Gb3 is associated with change in certain clinical parameters, such LVmass and development of white matter lesions or stroke. Thus, these results suggest a possible deleterious effect these antibodies on clinical efficacy of ERT, a finding that needs to be confirmed in a larger cohort. This emphasizes the urgent need to characterize the impact of anti-agalsidase antibodies on clinical efficacy of ERT in larger cohorts.

## References

[1] Schiffmann R, Warnock DG, Banikazemi M, Bultas J, Linthorst GE, et al (2009) Fabry disease: progression of nephropathy, and prevalence of cardiac and cerebrovascular events before enzyme replacement therapy. Nephrol Dial Transplant (7): 2102–11. 10.1093/ndt/gfp031 PMC2698092 19218538

[2] Vedder AC, Linthorst GE, van Breemen MJ, Groener JEM, Bemelman FJ, et al (2007) The Dutch Fabry cohort: diversity of clinical manifestations and Gb3 levels. J Inherit Metab Dis 30(1): 68–78. 17206462 10.1007/s10545-006-0484-8

[3] Eng CM, Guffon N, Wilcox WR, Germain DP, Lee P, et al (2001) Safety and efficacy of recombinant human alpha-galactosidase A--replacement therapy in Fabry's disease. N Engl J Med 345(1): 9–16. 11439963 10.1056/NEJM200107053450102

[4] Schiffmann R, Kopp JB, Austin HA, Sabnis S, Moore DF, et al (2001) Enzyme replacement therapy in Fabry disease: a randomized controlled trial. JAMA 285(21): 2743–2749. 11386930 10.1001/jama.285.21.2743

[5] Linthorst GE, Hollak CEM, Donker-Koopman WE, Strijland A, Aerts JMFG (2004) Enzyme therapy for Fabry disease: neutralizing antibodies toward agalsidase alpha and beta. Kidney Int 66(4): 1589–1595. 15458455 10.1111/j.1523-1755.2004.00924.x

[6] Bénichou B, Goyal S, Sung C, Norfleet AM, Obrien F (2009) A retrospective analysis of the potential impact of IgG antibodies to agalsidase β on efficacy during enzyme replacement therapy for Fabry disease. Mol Genet Metab 96(1): 4–12. 19022694 10.1016/j.ymgme.2008.10.004

[7] Hollak CEM, Linthorst GE (2009) Immune response to enzyme replacement therapy in Fabry disease: impact on clinical outcome? Mol Genet Metab. 96(1): 1–3. 10.1016/j.ymgme.2008.10.013 19062323

[8] Vedder AC, Breunig F, Donker-Koopman WE, Mills K, Young E, et al (2008) Treatment of Fabry disease with different dosing regimens of agalsidase: effects on antibody formation and GL-3. Mol Genet Metab. 94(3): 319–325. 10.1016/j.ymgme.2008.03.003 18424138

[9] Aerts JM, Groener JE, Kuiper S, Donker-Koopman WE, Strijland A, et al (2008) Elevated globotriaosylsphingosine is a hallmark of Fabry disease. Proc Natl Acad Sci 105(8): 2812–2817. 18287059 10.1073/pnas.0712309105 PMC2268542

[10] Rombach SM, Dekker N, Bouwman MG, Linthorst GE, Zwinderman AH, et al (2010) Plasma globotriaosylsphingosine: Diagnostic value and relation to clinical manifestations of Fabry disease. BBA - Molecular Basis of Disease. 9: 1–8. 10.1016/j.bbadis.2010.05.003 20471476

[11] Sanchez-Niño MD, Sanz AB, Carrasco S, Saleem MA, Mathieson PW, et al (2011) Globotriaosylsphingosine actions on human glomerular podocytes: implications for Fabry nephropathy. Nephrol Dial Transplant 26(6): 1797–802. 20504837 10.1093/ndt/gfq306

[12] van Breemen MJ, Rombach SM, Dekker N, Poorthuis BJ, Linthorst GE, et al (2011) Reduction of elevated plasma globotriaosylsphingosine in patients with classic Fabry disease following enzyme replacement therapy. Biochim Biophys Acta 1812(1): 70–76. 20851180 10.1016/j.bbadis.2010.09.007

[13] Smid BE, Rombach SM, Aerts JM, Kuiper S, Mirzaian M, et al (2011) Consequences of a global enzyme shortage of agalsidase beta in adult Dutch Fabry patients. Orphanet J Rare Dis 31 6(1): 69. 10.1186/1750-1172-6-69 PMC3219561 22041095

[14] Vedder AC, Linthorst GE, Houge G, Groener JEM, Ormel EE, et al (2007) Treatment of Fabry disease: outcome of a comparative trial with agalsidase alfa or beta at a dose of 0.2 mg/kg. PLoS ONE 2(7): e598. 17622343 10.1371/journal.pone.0000598 PMC1913555

[15] Levey AS, Bosch JP, Lewis JB, Greene T, Rogers N, et al (1999) A more accurate method to estimate glomerular filtration rate from serum creatinine: a new prediction equation. Modification of Diet in Renal Disease Study Group. Ann Intern Med 130(6): 461–470. 10075613 10.7326/0003-4819-130-6-199903160-00002

[16] Devereux RB, Alonso DR, Lutas EM, Gottlieb GJ, Campo E, et al (1986) Echocardiographic assessment of left ventricular hypertrophy: comparison to necropsy findings. Am J Cardiol 57(6): 450–458. 2936235 10.1016/0002-9149(86)90771-x

[17] Groener JEM, Poorthuis BJHM, Kuiper S, Helmond MTJ, Hollak CEM, et al (2007) HPLC for Simultaneous Quantification of Total Ceramide, Glucosylceramide, and Ceramide Trihexoside Concentrations in Plasma. Clin Chem. 53(4): 742–747. 10.1373/clinchem.2006.079012 17332150

[18] Bligh EG, Dyer WJ (1959) A rapid method of total lipid extraction and purification. Can J Biochem Physiol 37(8): 911–917. 13671378 10.1139/o59-099

[19] Young E, Mills K, Morris P, Vellodi A, Lee P et al. (2005) Is globotriaosylceramide a useful biomarker in Fabry disease? Acta Paediatr Suppl 94(447):51–4 10.1111/j.1651-2227.2005.tb02112.x 15895713

[20] Bekri S, Lidove O, Jaussaud R, Knebelmann B, Barbey F (2006) The role of ceramide trihexoside (globotriaosylceramide) in the diagnosis and follow-up of the efficacy of treatment of Fabry disease: a review of the literature. Cardiovasc Hematol Agents Med Chem. 4(4): 289–297. 10.2174/187152506778520718 17073606

[21] Wilcox WR, Linthorst GE, Germain DP, Feldt-Rasmussen U, Waldek S, et al (2012) Anti-α-galactosidase A antibody response to agalsidase beta treatment: Data from the Fabry registry. Mol Genet Metab 105(3): 443–9. 22227322 10.1016/j.ymgme.2011.12.006

[22] Mendelsohn NJ, Messinger YH, Rosenberg AS, Kishnani PS (2009) Elimination of antibodies to recombinant enzyme in Pompe's disease. N Engl J Med. 360(2): 194–195. 10.1056/NEJMc0806809 19129538

[23] Garman RD, Munroe K, Richards SM (2004) Methotrexate reduces antibody responses to recombinant human alpha-galactosidase A therapy in a mouse model of Fabry disease. Clin Exp Immunol 137(3): 496–502. 15320898 10.1111/j.1365-2249.2004.02567.x PMC1809149

[24] Lubanda J, Anijalg E, Bzdúch V, Thurberg B, Bénichou B, et al (2009) Evaluation of a low dose, after a standard therapeutic dose, of agalsidase beta during enzyme replacement therapy in patients with Fabry disease. Genet Med. 11(4): 256–64. 10.1097/GIM.0b013e3181981d82 19265719

[25] Banikazemi M, Bultas J, Waldek S, Wilcox WR, Whitley CB, et al (2007) Agalsidase-beta therapy for advanced Fabry disease: a randomized trial. Ann Intern Med 146(2): 77–86. 17179052 10.7326/0003-4819-146-2-200701160-00148

[26] Breunig F, Weidemann F, Strotmann J, Knoll A, Wanner C (2006) Clinical benefit of enzyme replacement therapy in Fabry disease. Kidney Int 69(7): 1216–1221. 16609685 10.1038/sj.ki.5000208

[27] Weideman F, Niemann M, Breunig F, Herrmann S, Beer M, et al (2009) Long-Term Effects of Enzyme Replacement Therapy on Fabry Cardiomyopathy. Circulation 119(4): 487–488. 10.1161/CIRCULATIONAHA.108.794529 19153271

[28] Germain DP, Waldek S, Banikazemi M, Bushinsky DA, et al (2007) Sustained, long-term renal stabilization after 54 months of agalsidase beta therapy in patients with Fabry disease. J Am Soc Nephrol. 18(5): 1547–1557. 10.1681/ASN.2006080816 17409312

[29] Schellekens H (2008) The immunogenicity of therapeutic proteins and the Fabry antibody standardization initiative. . Clinical Therapeutics. 30 Suppl BS50–1. 18395140 10.1016/s0149-2918(08)80041-0

